# Biomarkers identification in follicular fluid of women with OHSS by using UPLC-MS method

**DOI:** 10.3389/fendo.2023.1131771

**Published:** 2023-03-08

**Authors:** Ze Wu, Lanlan Fang, Boqun Liu, Qiongqiong Jia, Jung-Chien Cheng, Ying-Pu Sun

**Affiliations:** Center for Reproductive Medicine, Henan Key Laboratory of Reproduction and Genetics, The First Affiliated Hospital of Zhengzhou University, Zhengzhou, Henan, China

**Keywords:** OHSS, follicular fluid, estradiol, association analysis, biomarker, differentially changed metabolites

## Abstract

To figure out the differentially changed metabolites and disturbed pathways in follicular fluid (FF) of patients with OHSS in comparison to the control group undergoing *in vitro* fertilization (IVF), we conducted this metabolomic analysis between two groups, the OHSS group included 30 patients treated with oocyte retrieval and developed OHSS in the next 7-14 days, while another 30 patients without OHSS tendency were selected as the control group. The FF samples were obtained during the process of oocyte retrieval. FF samples were analyzed using ultra-high liquid chromatography-tandem mass spectrometry (UPLC-MS). The results identified a total of 59 differentially changed metabolites, including 33 decreased metabolites (P < 0.01) and 26 increased metabolites (P < 0.01) in FF of OHSS compared with the control group. 12 metabolites could be the most valuable biomarkers for OHSS based on ROC results. Our correlation analyses showed that deoxyinosine levels were found positively correlated with serum estradiol (E2) levels in OHSS patients, while L-isoleucine, pyruvic acid, maleamate, and arachidonic acid were found to be positively correlated with the number of retrieved oocytes. Furthermore, 4-hydroxyphenylacetaldehyde, deoxycorticosterone, creatinine, and creatine were found to be negatively associated with serum E2 levels, while 4-hydroxyphenylacetaldehyde, L-carnitine, isovaleric acid and L-2-hydroxyglutaric acid were negatively related with the number of oocytes retrieved in OHSS patients. Taken together, our study provides better identification of OHSS FF metabolic dynamics, suggesting the metabolic compounds can be used as valuable predictors or treatment targets of OHSS.

## Introduction

Ovarian hyperstimulation syndrome (OHSS) is one of the most severe complications that occur in patients treated with controlled ovarian stimulation (COS) and *in vitro* fertilization/intracytoplasmic sperm injection-embryo transfer (IVF/ICSI-ET). The main manifestations of OHSS are enlarged ovaries, increased blood vessel permeability, the third body cavity effusion such as pleural effusion and peritoneal effusion, and other related pathophysiological changes (1). In severe cases, OHSS can be life-threatening.

The etiology of OHSS remains largely unknown. Human chorionic gonadotropin (hCG) injection is the key step to stimulating the ovary to release a large number of vasoactive substances, such as vascular endothelial growth factor (VEGF), histamine, prostaglandin, variable cytokines, inflammatory mediators, ovarian renin-angiotensin system factors and pigment epithelium-derived factor. Particularly, VEGF is the most important factor that stimulates vascular endothelial proliferation, which promotes angiogenesis and increases vascular permeability in patients with OHSS (1–3). Age, body mass index (BMI), antral follicle count (AFC), serum anti-Mullerian hormone (AMH) levels, and serum estradiol (E2) levels can be used to predict OHSS (4, 5). Additionally, our previous studies revealed several novel biomarkers that participated in OHSS and may be used as potential treatment targets, such as GDF8, melatonin, TGF-β1, amphiregulin, and sprouty2 (6–10).

Metabolomics, a technological tool for analyzing compounds, such as amino acids, carbohydrates, and lipids, will provide a collection of distinct metabolites and the integration of their profiles for medical use, such as obesity, diabetes, cardiovascular diseases, cancer, and neurodegenerative diseases (11). More importantly, metabolomics are extensively used in a variety of body fluids including cerebrospinal fluid (12), pleural effusion (13), and ascites (14) to identify potential markers. Since inflammation conditions are related to high vascular permeability and effusion, which are similar to OHSS, we aim to figure out whether metabolomics could predict OHSS or be used as new markers to prevent OHSS (15).

In terms of reproduction, follicular fluid (FF) is an important and accessible sample for evaluating the ovary microenvironment under reproductive physiology and pathology condition. FF components analysis can provide unique information on metabolic or other microenvironment changes in the ovary. It was reported that the metabolomic approach is a powerful tool for revealing biochemical predictors of oocyte quality in FF (16). Furthermore, FF metabolic variations were also discovered in endometriosis, polycystic ovary syndrome (PCOS), diminished ovarian reserve, and premature ovarian insufficiency (POI) patients (17–21). Recently, one lipidomic analysis was conducted on FF of OHSS, and the results showed that lipid metabolism changed and 10 lipids were regarded as differentially changed metabolites (22). OHSS is regarded as a characteristic syndrome in the process of COS, and FF is waste of oocytes retrieved process. Moreover, the relationship of OHSS is closer with FF components than serum. In addition, OHSS occurs after oocytes are retrieved, we therefore consider that FF biomarkers testing is a less invasive and more valuable method than serum for OHSS prediction. Therefore, it is urgent to explore the changes in various metabolic components, such as sugar, amino acid, and lipid derivatives in the FF of OHSS patients and establish new diagnostic and treatment markers for OHSS.

## Materials and methods

### Patients

This study included 30 OHSS patients and 30 control patients who received IVF/ICSI-ET from the Reproductive Medicine Center of the First Affiliated Hospital of Zhengzhou University from July 27, 2021, to October 07, 2021. Informed consent was obtained from all patients. The study received approval from the Ethics Committee of Scientific Research and Clinical Trial (Ethical review number: 2022-KY-252) of the First Affiliated Hospital of Zhengzhou University. The study received approval and was carried out in accordance with the approved guidelines from the First Affiliated Hospital of Zhengzhou University Research Ethics Board. Amongst all patients, the included criteria were as follows: age between 20 and 35, BMI between 15 and 31, normal menstrual cycle, with male infertility or tubal factor infertility, and without complications such as diabetes, thyroid malfunctions, et al. The exclusion criteria were as follows: endometriosis, chromosome conditions, hydrosalpinx, or diminished ovarian reserve. The OHSS diagnostic criteria is defined as: (a) Mild OHSS: abdominal bloating, mild abdominal pain, ovarian size usually < 8 cm; (b) Moderate OHSS: moderate abdominal pain, nausea ± vomiting, ultrasound evidence of ascites, ovarian size usually 8 to 12 cm; (c) Severe OHSS: clinical ascites (occasionally pleural effusion), oliguria, hemoconcentration hematocrit (> 45%), hypoproteinemia, ovarian size usually > 12 cm; (d) Critical OHSS: tense ascites or large pleural effusion, hematocrit (> 55%), white cell count > 25 000, oligouria/anuria, thromboembolism, acute respiratory distress syndrome (23, 24). Upon gonadotropin (Gn) and hCG administration and the followed oocytes retrieval, 30 patients diagnosed with OHSS were recruited, and they did not undergo the following embryo transfer. Another 30 patients undergoing embryo transfer without OHSS tendency were randomly selected as the control group, which were matched by age, BMI, serum levels of LH, progesterone on hCG day, total gonadotropin, gonadotropin treatment days, and fertility cause with OHSS group, as shown in **Table 1**.

**Table 1:** Clinical information for OHSS patients and the control group.

	Non-OHSS N=30	OHSS N=30	p-value
Maternal age, years	29.100 ± 3.067	29.300 ± 3.612	
BMI, kg/m²	21.795 ± 3.411	23.353 ± 3.197	0.073
AMH, ng/mL	2.627 ± 0.932	5.082 ± 2.511	<0.01
Infertility cause			
Male factor, N (%)	1 (3.3%)	3 (10%)	
Tubal factor, N (%)	15 (50%)	16 (53.3%)	
Unexplained, N (%)	14 (46.7%)	11 (36.7%)	
Total Gn used (IU)	2329.567 ± 838.507	1936.250 ± 753.629	0.061
Gn treatment days	13.267 ±1.893	13.467 ± 1.776	
E_2_ on hCG day, pg/mL	3224.100 ± 1427.727	5756.933 ± 2331.728	<0.01
Oocytes retrieved day			
Diameter of left ovary, cm	3.805 ± 0.526	4.558 ± 0.669	<0.01
Diameter of right ovary, cm	3.917 ± 0.644	4.766 ± 0.885	<0.01
No. of oocytes retrieved	11.467 ± 1.737	15.400 ± 3.212	<0.01
Embryo transfer day			
Diameter of left ovary, cm	4.866 ± 0.818	6.511 ± 0.977	<0.01
Diameter of right ovary, cm	5.057 ± 0.948	6.683 ±1.314	<0.01

The data were presented as mean ± SEM. BMI, body mass index; AMH, anti-mullerian hormone; Gn, gonadotropin; E_2_, estradiol; HCG, human chorionic gonadotropin; cm, centimeter.

### COH protocol

The patients were treated with gonadotropin-releasing hormone agonist (GnRH-a) pituitary downregulation protocol. On the second day of menstruation, the GnRH-a (3.75 mg) (Merck, Darmstadt, Germany), was administered subcutaneously. Approximately 30 days after GnRH agonist injection, recombinant FSH (Gonal-F; Merck, Darmstadt, Germany) was administered daily at a dosage of 100-300 IU. When at least three follicles had reached 18 mm, final oocyte maturation was conventionally induced by 6,500 IU recombinant human chorionic gonadotropin and 2,000 IU urinary hCG (Livzon, Zhuhai, China). Oocyte retrieval was scheduled approximately 37 h after hCG injection by transvaginal ultrasound-guided follicular aspiration.

### Follicular fluid collection

Only the follicular fluid aspirate without blood or flushing solution was used for analysis. The FF samples of the same patient were combined as one for analysis. After 10 min of centrifugation at 1200rpm, the supernatant was stored at −80°C until further analysis.

### Quality control sample standard curve correction solution

Firstly, a pooled FF sample from 3 healthy controls and 3 OHSS patients was used as quality control (QC) and underwent the same metabolites extraction procedures. We transfer 5, 10, 50, 100, 200, and 300 μL of QC sample into centrifuge tubes. Add H_2_O, mixed internal standard solution, and methanol. Then we vortex the six QC samples, centrifuge at 4 °C for 10 min at 12 000 rpm, and then transfer all the supernatant from each sample into another 2 mL centrifuge tube. Samples were concentrated to dryness in a vacuum. The samples were dissolved with 150 μL of 80% methanol solution (-20°C), and centrifuged at 4°C for 10 min at 12 000 rpm again to obtain the supernatant for LC-MS.

### Metabolites extraction

All the samples were thawed at 4 °C (the insufficient samples are reduced to an equal scale); Thereafter, 100 μl FF were transferred into 2 mL centrifuge tube (samples with a sample size less than 50 µL were extracted by half of the experimental system, but the resolution system remained unchanged); 100 µL of mixed internal standard solution and 400 µL of methanol (-20 °C) were added, then vortexed for 60 s. Then the samples were centrifuged at 4 °C for 10 min at 12000 rpm, and 500 µL of the supernatant from each sample were transferred into another 2 mL centrifuge tube. Samples were concentrated to dryness in a vacuum and dissolved with 150 μL of 80% methanol solution and centrifuged at 4 °C for 10 min at 12000 rpm again to obtain the supernatant for LC-MS. Then the 20 µL QC samples were taken and used for LC-MS detection (these QC samples were used to monitor deviations of the analytical results from these pool mixtures and compare them to the errors caused by the analytical instrument itself).

### UPLC-MS process

#### Chromatographic conditions

Chromatographic separation was accomplished in a Thermo Vanquish system equipped with an ACQUITY UPLC^®^ HSS T3 (150×2.1 mm, 1.8 µm, Waters) column maintained at 40 °C. The temperature of the autosampler was 8 °C. Gradient elution of analysis was carried out with 0.1% formic acid in water (A2) and 0.1% formic acid in acetonitrile (B2) or 5 mM ammonium formate in water (A3) and acetonitrile (B3) at a flow rate of 0.25 mL/min. Injection of 2 μL of each sample was done after equilibration. An increasing linear gradient of solvent B2/B3 (v/v) was used as follows: 0~1 min, 2% B2/B3; 1~9 min, 2%~50% B2/B3; 9~12 min, 50%~98% B2/B3; 12~13.5 min, 98% B2/B3; 13.5~14 min, 98%~2% B2/B3; 14~20 min, 2% B2-positive model (14~17 min, 2% B3-negative model).

#### Mass spectrum conditions

The ESI-MSn experiments were executed on the Thermo Q Exactive mass spectrometer with the spray voltage of 3.5 kV and -2.5 kV in positive and negative modes, respectively. Sheath gas and auxiliary gas were set at 30 and 10 arbitrary units, respectively. The capillary temperature was 325 °C. The analyzer scanned over a mass range of m/z 81-1 000 for the full scan at a mass resolution of 70 000. Data-dependent acquisition (DDA) MS/MS experiments were performed with HCD scan. The normalized collision energy was 30 eV. Dynamic exclusion was implemented to remove some unnecessary information in MS/MS spectra.

#### Data analysis

Data of UPLC-MS were processed through Progenesis QI 2.0 software and Ezinfo 3.0 (Waters), which performed automatic baseline correction, alignment, and peak peaking. The peaks of missing values were removed by the 80% rule (25). The elected peak index with accurate m/z and fragment information were submitted to online library search, including Metlin (http://metlin.scripps.edu), MoNA (https://mona.fiehnlab.ucdavis.edu//), and HMDB. Multivariate analysis, Student’s t-test, and correlation analysis between IVF clinical data and differentially changed metabolites were performed using STATA software (version 15.0, Stata Corporation, College Station, TX, USA). The heat map and ANOVA result were constructed by R (version 3.3.1). The data were presented as mean **±** SEM. A significant difference was defined as p < 0.01.

## Results

### Clinical characteristics of patients

The clinical characteristics of OHSS and control patients are shown in **Table 1**. Among the included 30 OHSS patients, 1 (3.33%) showed mild, 5 (16.67%) showed moderate, 6 (20%) showed severe, and 18 (60%) showed critical OHSS, because of the less number of mild OHSS, we do not analyze differentially changed metabolites between different OHSS classification. AMH, serum E2 concentration on the day of hCG administration, and the number of oocytes retrieved (No. of oocytes retrieved) were significantly higher in OHSS, which was consistent with previous reports (26, 27), indicating higher responsiveness in OHSS. No statistical differences were observed in age, BMI, total gonadotropin usage as well as gonadotropin treatment days.

### Principal component analysis, partial least squares-discriminate analysis, and orthogonal projections to latent structures discriminant analysis results

**Figure 1:**
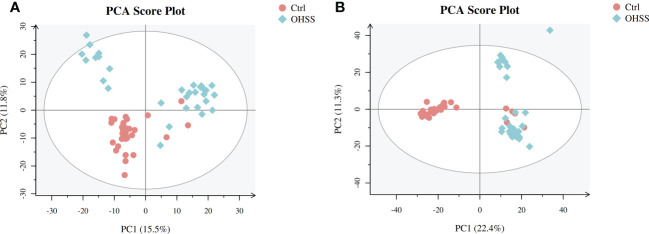
PCA score plots of the control and OHSS groups based on follicular fluid spectral data. Follicular fluid metabolomics profiles examined by PCA demonstrated a differentiation between the control and OHSS groups. Score plot of the first two principal components (PC1, PC2) from PCA model obtained when comparing the control group and OHSS group: **(A)** ESI positive mode; **(B)** ESI negative mode. One point stands for one subject, red circles represent the control, blue diamonds represent OHSS.

**Figure 2:**
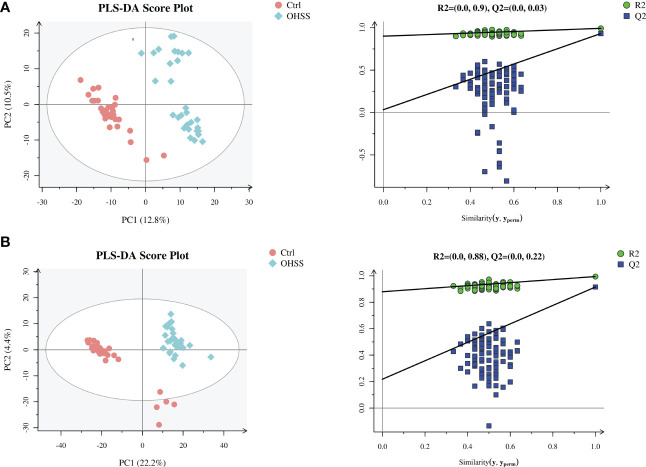
PLS-DA score plots of different groups based on follicular fluid spectral data of ESI ion modes. Follicular fluid metabolomics profiles examined by PLS-DA demonstrated a clear differentiation between the control and OHSS groups. Score plot of the first two principal components (PC1, PC2) from PLS-DA model obtained when comparing the control group and OHSS group: **(A)** ESI positive mode, model parameters: R2Y=0.992, Q2 = 0.931; **(B)** ESI negative mode, model parameters: R2Y=0.994, Q2 = 0.915. One point stands for one subject, red circles represent the control, blue diamonds represent OHSS.

**Figure 3:**
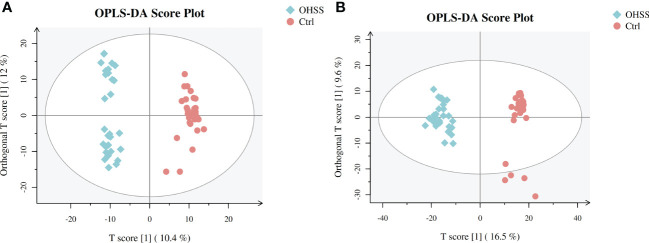
OPLS-DA score plots of two ESI modes based on follicular fluid metabolic profiling of OHSS group and the control group. **(A)** ESI positive mode, model parameters: R2Y=0.977, Q2 = 0.839; **(B)** ESI negative mode, model parameters: R2Y=0.95, Q2 = 0.827. One point stands for one subject, red circles represent the control, blue diamonds represent OHSS.

We first conducted Principal component analysis (PCA) analysis, the separation was insufficient with overlapping of samples from the control and OHSS groups in both Electron Spray Ionization (ESI) models (**Figure 1**). Partial least squares-discriminate analysis (PLS-DA), which is a commonly recognized dimension-reduction technique, summarized the largest group-wise difference in complex multivariate data. The PLS-DA score plots showed that the two FF groups presented clearly separated trends in both ESI models (**Figure 2**). A significant variation of OHSS FF samples was observed when compared to the control group, which indicated that metabolic fingerprints difference did exist between OHSS and the control groups. To further identify the differentially changed metabolites, the orthogonal projections to latent structures discriminant analysis (OPLS-DA) was applied. A more obvious separation of the two groups was observed in both ESI models (**Figure 3**).

### Differentially changed metabolites in FF between OHSS and the control groups

**Figure 4:**
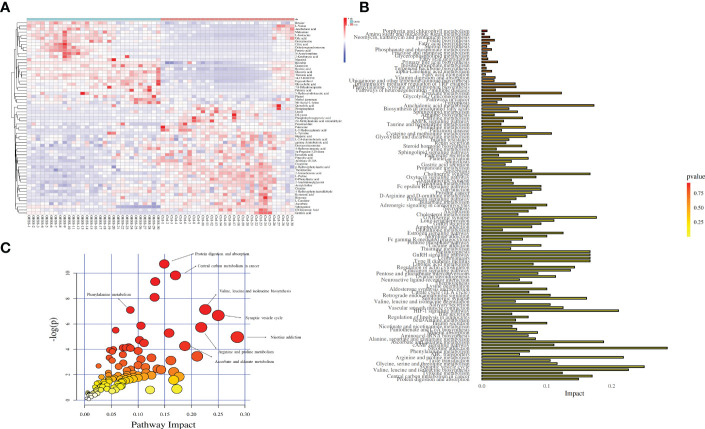
Thermogram and disturbed pathway for OHSS. **(A)** Thermogram for 59 differentially changed metabolites (Red: control women; blue: women with OHSS). **(B)** Bubble chart for disturbed pathways in OHSS, each point represents one metabolic pathway; the size of dot and shades of color are positive correlation with the impaction of metabolic pathway. **(C)** Histogram chart for disturbed pathways in OHSS.

After database identification, 1755 metabolites were identified in ESI-positive mode, and 2436 metabolites were identified in ESI-negative mode. After applying more rigorous filtering strategies, 59 metabolites obtained with corrected p<0.01 are listed in **Table 2**. Additionally, a thermogram was applied to illustrate their corresponding contents (**Figure 4A**), from which the different patterns of the two groups can also be visually observed. Alterations of amino acid metabolites were observed in FF from two sample groups. Levels of D-phenyllactic acid, L-proline, hippuric acid, (S)-methylmalonic acid semialdehyde, kynurenic acid, pipecolic acid, putrescine, creatinine, L-tyrosine, gentisic acid, norepinephrine, gamma-aminobutyric acid, L-2,4-diaminobutyric acid, creatine, and 4-hydroxyphenylacetaldehyde decreased in the FF from OHSS patients, whereas levels of betaine, L-isoleucine, N-acetylornithine, 5-hydroxyindoleacetic acid, L-valine, and N6-acetyl-L-lysine increased.

**Table 2:** Differentially changed metabolites that varied in OHSS FF with significant differences.

	Super pathway	Subpathway	Class	Sub-class	VIP	Mean Ctrl	SD Ctrl	Mean OHSS	SD OHSS	-log(p)	Impact	Fold Change OHSS/Ctrl
D-Phenyllactic acid	Amino acid	Phenylalanine metabolism	Phenylpropanoic acids	NULL	2.218	0.684	0.449	0.036	0.139	7.102	0.085	0.052
Betaine	Amino acid	Glycine, serine and threonine metabolism	Carboxylic acids and derivatives	Amino acids, peptides, and analogues	1.986	0.623	0.190	1.117	0.393	8.101	0.131	1.792
L-Proline	Amino acid	Arginine and proline metabolism	Carboxylic acids and derivatives	Amino acids, peptides, and analogues	1.939	1.182	0.467	0.611	0.242	5.732	0.218	0.516
L-Isoleucine	Amino acid	Valine, leucine and isoleucine degradation	Carboxylic acids and derivatives	Amino acids, peptides, and analogues	1.718	0.207	0.199	0.628	0.251	3.119	0.051	3.040
Hippuric acid	Amino acid	Phenylalanine metabolism	Benzene and substituted derivatives	Benzoic acids and derivatives	1.547	1.780	0.892	0.990	0.467	7.102	0.085	0.556
N-Acetylornithine	Amino acid	Arginine biosynthesis	Carboxylic acids and derivatives	Amino acids, peptides, and analogues	1.484	0.442	0.239	4.047	4.845	1.029	0.019	9.174
(S)-Methylmalonic acid semialdehyde	Amino acid	Valine, leucine and isoleucine degradation	Organooxygen compounds	Carbonyl compounds	1.462	11.728	14.955	0.799	0.868	3.119	0.051	0.068
Kynurenic acid	Amino acid	Tryptophan metabolism	Quinolines and derivatives	Quinoline carboxylic acids	1.408	1.102	0.589	0.647	0.286	2.644	0.063	0.587
Pipecolic acid	Amino acid	Lysine metabolism	Carboxylic acids and derivatives	Amino acids, peptides, and analogues	1.401	0.984	0.462	0.583	0.350			0.593
Putrescine	Amino acid	Arginine and proline metabolism	NULL	NULL	1.320	0.700	0.517	0.346	0.189	5.732	0.218	0.495
Creatinine	Amino acid	Arginine and proline metabolism	Carboxylic acids and derivatives	Amino acids, peptides, and analogues	1.276	1.931	1.708	0.366	0.827	5.732	0.218	0.190
L-Tyrosine	Amino acid	Tyrosine metabolism	Carboxylic acids and derivatives	Amino acids, peptides, and analogues	1.270	0.541	0.406	0.147	0.498	9.339	0.132	0.272
Gentisic acid	Amino acid	Tyrosine metabolism	Benzene and substituted derivatives	Benzoic acids and derivatives	1.258	5.548	4.703	2.501	1.771	9.339	0.132	0.451
Norepinephrine	Amino acid	Tyrosine metabolism	Phenols	Benzenediols	1.251	1.883	2.287	0.435	0.740	9.339	0.132	0.231
gamma-Aminobutyric acid	Amino acid	Alanine, aspartate and glutamate metabolism	Carboxylic acids and derivatives	Amino acids, peptides, and analogues	1.207	1.163	0.477	0.804	0.398	4.186	0.082	0.692
L-2,4-diaminobutyric acid	Amino acid	Glycine, serine and threonine metabolism	Carboxylic acids and derivatives	Amino acids, peptides, and analogues	1.185	1.077	0.374	0.820	0.260	8.101	0.131	0.761
5-Hydroxyindoleacetic acid	Amino acid	Tryptophan metabolism	Indoles and derivatives	Indolyl carboxylic acids and derivatives	1.152	0.692	0.419	4.250	6.496	2.644	0.063	6.135
L-Valine	Amino acid	Valine, leucine and isoleucine degradation	Carboxylic acids and derivatives	Amino acids, peptides, and analogues	1.122	0.570	0.973	1.352	0.556	3.119	0.051	2.370
Creatine	Amino acid	Arginine and proline metabolism	Carboxylic acids and derivatives	Amino acids, peptides, and analogues	1.067	1.107	0.512	0.800	0.338	5.732	0.218	0.723
N6-Acetyl-L-lysine	Amino acid	Lysine degradation	Carboxylic acids and derivatives	Amino acids, peptides, and analogues	1.025	1.160	0.368	1.644	0.941	2.690	0.026	1.416
4-Hydroxyphenylacetaldehyde	Amino acid	Tyrosine metabolism	Benzene and substituted derivatives	Phenylacetaldehydes	1.019	0.661	0.319	0.448	0.315	9.339	0.132	0.677
L-Carnitine	Lipid	Carnitine metabolism	Organonitrogen compounds	Quaternary ammonium salts	1.970	1.018	0.529	0.364	0.250			0.358
14,15-DiHETrE	Lipid	Arachidonic acid metabolism	Fatty Acyls	Eicosanoids	1.903	0.181	0.213	0.623	0.168	0.870	0.173	3.448
Sphinganine	Lipid	SphingoLipid	Organonitrogen compounds	Amines	1.850	2.918	0.179	2.385	0.496	1.386	0.069	0.817
Dehydroepiandrosterone	Lipid	Steroid hormone biosynthesis	Steroids and steroid derivatives	Androstane steroids	1.609	0.320	0.560	2.954	2.187	1.240	0.061	9.259
Bovinic acid	Lipid	Linoleic acid metabolism	Fatty Acyls	Lineolic acids and derivatives	1.480	0.072	0.055	0.136	0.031	2.326	0.079	1.887
L-2-Hydroxyglutaric acid	Lipid	Hydroxy fatty acids	Fatty Acyls	Fatty acids and conjugates	1.478	3.796	2.624	1.025	0.726			0.270
Ergocalciferol	Lipid	Steroid biosynthesis	Steroids and steroid derivatives	Vitamin D and derivatives	1.458	0.087	0.056	0.181	0.076	0.395	0.007	2.075
Isovaleric acid	Lipid	Saturated fatty acids	Fatty Acyls	Fatty acids and conjugates	1.368	0.255	0.204	0.095	0.124	0.886	0.042	0.372
Acetylcholine	Lipid	GlycerophosphoLipid	Organonitrogen compounds	Quaternary ammonium salts	1.339	1.051	0.426	0.692	0.348	0.457	0.007	0.658
5a-Pregnane-3,20-dione	Lipid	Steroid hormone biosynthesis	Steroids and steroid derivatives	Pregnane steroids	1.282	1.064	0.545	0.694	0.243	1.240	0.061	0.652
Glycocholic acid	Lipid	Primary bile acid biosynthesis	Steroids and steroid derivatives	Bile acids, alcohols and derivatives	1.148	0.151	0.207	0.699	0.736	0.517	0.024	4.651
Palmitic acid	Lipid	Fatty acid biosynthesis	Fatty Acyls	Fatty acids and conjugates	1.079	0.198	0.255	0.789	0.853	0.395	0.011	3.984
Arachidonic acid	Lipid	Arachidonic acid metabolism	Fatty Acyls	Fatty acids and conjugates	1.030	0.332	0.633	0.911	0.670	0.870	0.173	2.740
Methyl jasmonate	Lipid	alpha-Linolenic acid metabolism	Fatty Acyls	Lineolic acids and derivatives	1.029	0.472	0.371	0.765	0.482	0.559	0.015	1.621
Deoxycorticosterone	Lipid	Steroid hormone biosynthesis	Steroids and steroid derivatives	Hydroxysteroids	1.019	0.992	0.405	0.731	0.372	1.240	0.061	0.736
4-Hydroxycinnamic acid	Xenobiotics	Food Component/Plant	Cinnamic acids and derivatives	Hydroxycinnamic acids and derivatives	1.031	2.234	0.879	1.567	1.068			0.702
Theobromine	Xenobiotics	Caffeine metabolism	Imidazopyrimidines	Purines and purine derivatives	2.086	2.129	0.451	1.408	0.373	1.065	0.063	0.661
Antibiotic JI-20A	Xenobiotics	Neomycin, kanamycin and gentamicin biosynthesis	NULL	NULL	1.586	2.933	1.517	1.411	1.104	0.233	0.012	0.481
Phenol	Xenobiotics	Aminobenzoate degradation	NULL	NULL	1.401	0.563	0.289	1.078	0.687			1.916
Benzoate	Xenobiotics	Benzoate degradation	NULL	NULL	1.389	0.920	1.053	0.117	0.519			0.127
Guanosine	Nucleotide	Purine metabolism	Purine nucleosides	NULL	1.673	0.088	0.127	0.444	0.255	1.315	0.022	5.051
Uracil	Nucleotide	Pyrimidine metabolism	Diazines	Pyrimidines and pyrimidine derivatives	1.614	10.002	9.091	1.923	3.460	1.047	0.067	0.192
Deoxyinosine	Nucleotide	Purine metabolism	Purine nucleosides	Purine 2'-deoxyribonucleosides	1.553	0.135	0.224	1.142	0.888	1.315	0.022	8.475
Uric acid	Nucleotide	Purine metabolism	Imidazopyrimidines	Purines and purine derivatives	1.547	1.780	0.892	0.990	0.467	1.315	0.022	3.247
Pseudouridine	Nucleotide	Pyrimidine metabolism	Nucleoside and nucleotide analogues	NULL	1.017	0.644	0.385	0.311	0.377	1.047	0.067	0.483
Bilirubin	Cofactors and Vitamins	Porphyrin and chlorophyll metabolism	Azacyclic compounds	Bilirubins	1.568	0.334	0.094	0.439	0.002	0.064	0.008	1.314
Threonic acid	Cofactors and Vitamins	Ascorbate and aldarate metabolism	Organooxygen compounds	Carbohydrates and carbohydrate conjugates	2.026	0.206	0.281	1.049	0.338	4.261	0.188	5.076
7,8-Dihydroneopterin	Cofactors and Vitamins	Folate biosynthesis	Pteridines and derivatives	Pterins and derivatives	1.394	0.155	0.155	0.557	0.403	0.395	0.019	3.610
Maleamate	Cofactors and Vitamins	Nicotinate and nicotinamide metabolism	NULL	NULL	1.302	0.179	0.259	0.594	0.416	3.939	0.047	3.322
Ascorbate	Cofactors and Vitamins	Ascorbate and aldarate metabolism	NULL	NULL	1.171	1.281	0.498	0.927	0.394	4.261	0.188	0.724
Quinolinic acid	Cofactors and Vitamins	Nicotinate and nicotinamide metabolism	Pyridines and derivatives	Pyridinecarboxylic acids and derivatives	1.073	25.144	33.408	6.087	17.654	3.939	0.047	0.242
Gluconic acid	Carbohydrate	Pentose phosphate pathway	Organooxygen compounds	Carbohydrates and carbohydrate conjugates	1.751	0.223	0.257	0.754	0.293	1.955	0.045	3.378
D-Glucuronic Acid	Carbohydrate	Pentose and glucuronate interconversions	NULL	NULL	1.564	1.228	0.714	0.615	0.289	2.422	0.086	0.501
Pyruvic acid	Carbohydrate	Pyruvate metabolism	Keto acids and derivatives	Alpha-keto acids and derivatives	1.357	0.616	1.074	4.871	4.617	0.800	0.122	7.937
Mannitol	Carbohydrate	Fructose and mannose metabolism	Organooxygen compounds	Carbohydrates and carbohydrate conjugates	1.352	0.142	0.135	0.848	0.780	0.435	0.008	5.988
2-Ketobutyric acid	Carbohydrate	Propanoate metabolism	Keto acids and derivatives	Short-chain keto acids and derivatives	1.340	0.971	0.347	4.963	6.092	1.466	0.069	5.102
D-Lyxose	Carbohydrate	Pentose and glucuronate interconversions	NULL	NULL	1.181	28.487	47.640	1.602	3.215	2.422	0.086	0.056
Citric acid	Carbohydrate	Citrate cycle (TCA cycle)	Carboxylic acids and derivatives	Tricarboxylic acids and derivatives	1.021	0.144	0.158	1.084	1.505	2.918	0.072	7.519

Levels of lipid metabolites, L-carnitine, sphinganine, L-2-hydroxyglutaric acid, isovaleric acid, acetylcholine, 5a-pregnane-3,20-dione, and deoxycorticosterone decreased in the FF from OHSS patients, however, 14,15-DiHETrE, dehydroepiandrosterone, bovinic acid, ergocalciferol, glycocholic acid, palmitic acid, arachidonic acid, and methyl jasmonate increased. Xenobiotics metabolite, phenol increased in the FF from OHSS patients, while antibiotic JI-20A, benzoate, theobromine, and 4-Hydroxycinnamic acid decreased. Irregular variations were also observed in other metabolite classes such as nucleotide, cofactors, vitamins, and carbohydrate.

### Metabolomic pathway analysis

In this study, functional enrichment analyses were used to figure out the biological roles of differentially changed metabolites. After the Kyoto Encyclopaedia of Genes and Genomes (KEGG) enrichment analysis and Metabolomics Pathway Analysis (MetPA), the results showed that the 59 metabolites were mainly centered on nicotine addiction (impact = 0.286), synaptic vesicle cycle (impact = 0.250), valine, leucine and isoleucine biosynthesis (impact = 0.226), arginine and proline metabolism (impact = 0.218), and ascorbate and aldarate metabolism (impact = 0.188). Other pathways, including central carbon metabolism in cancer, protein digestion and absorption, and phenylalanine metabolism were disturbed between OHSS and normal females (**Figures 4B, C**).

### Potential diagnostic predictors for OHSS according to the receiver operating characteristic curve results

**Figure 5:**
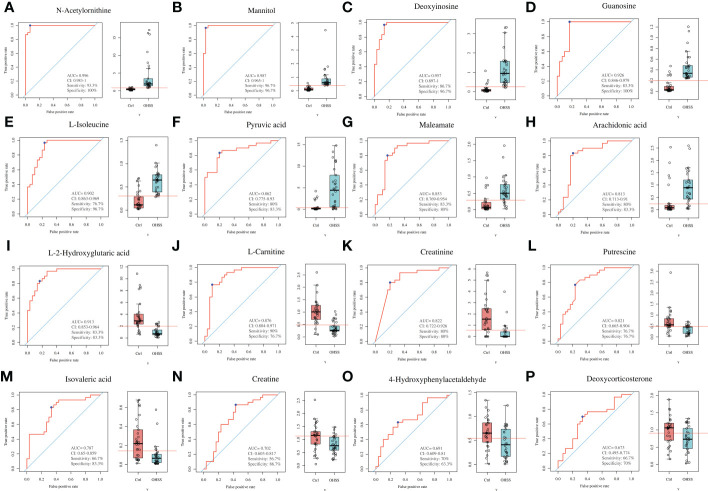
ROC curves of differentially changed metabolites in the FF of OHSS patients. **(A)** N-acetylornithine. **(B)** Mannitol. **(C)** Deoxyinosine. **(D)** Guanosine. **(E)** L-isoleucine. **(F)** Pyruvic acid. **(G)** Maleamate. **(H)** Arachidonic acid. **(I)** L-2-hydroxyglutaric acid. **(J)** L-carnitine. **(K)** Creatinine. **(L)** Putrescine. **(M)** Isovaleric acid. **(N)** Creatine. **(O)** 4-Hydroxyphenylacetaldehyde. **(P)** Deoxycorticosterone.

The receiver operating characteristic curve (ROC) curve analysis was used to identify valuable diagnostic markers for OHSS. As a result, although the area under the curves (AUC) of 59 differentially changed metabolites in our study showed that all of these values were more than 0.5, 16 altered metabolites with AUC ranging from 0.6 to 1 showed the best diagnostic accuracy for OHSS, including 8 increased metabolites (**Figures 5A–H**) and 8 decreased metabolites (**Figures 5I–P**) in OHSS compared with the control group. Among the increased metabolites in OHSS, AUC of N-acetylornithine, mannitol, deoxyinosine, guanosine, L-isoleucine, pyruvic acid, maleamate, and arachidonic acid were 0.996, 0.987, 0.957, 0.926, 0.902, 0.862, 0.853, and 0.813, respectively. Regarding the decreased metabolites in OHSS, AUC of L-2-hydroxyglutaric acid, L-carnitine, creatinine, putrescine, isovaleric acid, creatine, 4-hydroxyphenylacetaldehyde, and deoxycorticosterone were 0.913, 0.876, 0.822, 0.821, 0.787, 0.702, 0.691, and 0.673 respectively. We then collected 12 of the 16 metabolites with AUC ranging from 0.8 to 1.0 as potential diagnostic and treatment markers of OHSS.

### Correlation between detected metabolites and clinical characteristics

**Figure 6:**
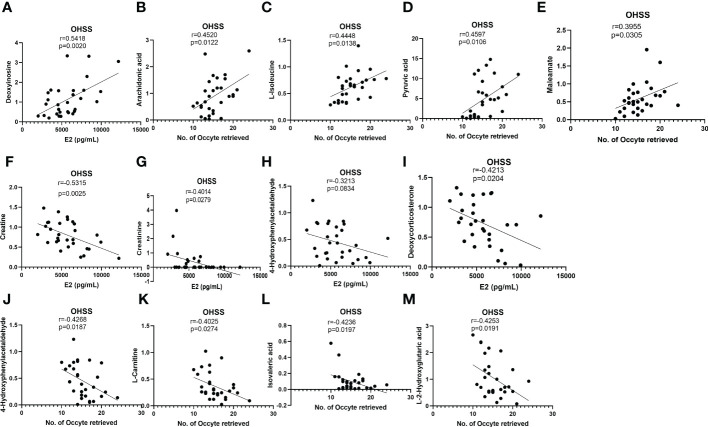
Correlation analyses between differentially changed metabolites and E2 or No. of oocytes retrieved in OHSS. **(A)** Deoxyinosine positively associated with E2 in OHSS. **(B)** arachidonic acid, **(C)** L-isoleucine, **(D)** pyruvic acid, and **(E)** maleamate positively associated with No. of oocytes retrieved in OHSS. **(F)** creatine, **(G)** creatinine, **(H)** 4-hydroxyphenylacetaldehyde, and **(I)** deoxycorticosterone negatively associated with E2 in OHSS. **(J)** 4-hydroxyphenylacetaldehyde, **(K)** L-carnitine, **(L)** isovaleric acid, and **(M)** L-2-hydroxyglutaric acid negatively associated with No. of oocytes retrieved in OHSS.

E2 on hCG day and the number of retrieved oocytes were important predictors of OHSS (27, 28). To explore the underlying correlation between FF differentially changed metabolites and patients’ pathological status, the Pearson correlation analysis was used to analyze the relationship among 16 differentially changed metabolites with potential diagnostic value and clinical features such as serum E2 levels on hCG day, and the No. of retrieved oocytes. Deoxyinosine levels were found positively correlated with E2 in OHSS patients. In the meantime, L-isoleucine, pyruvic acid, maleamate, and arachidonic acid were found to be positively correlated with the No. of oocytes retrieved (**Figures 6A–E**). However, 4-hydroxyphenylacetaldehyde, deoxycorticosterone, creatinine, and creatine were found to be negatively associated with serum E2 levels (**Figures 6F–I**). 4-hydroxyphenylacetaldehyde, L-carnitine, isovaleric acid, and L-2-hydroxyglutaric acid were negatively related with the No. of oocytes retrieved in OHSS patients (**Figures 6J–M**). Interestingly, the differentially changed metabolites positively correlated with E2 were also positively correlated with the number of retrieved oocytes and vice versa, although no statistical significance was found.

## Discussion

Since FF is the most important microenvironment supporting follicle development, oocyte maturation, and ovulation, the studies of FF components are urgently needed to reveal the regulation mechanism of reproductive physiology and pathology activities (29). FF compounds are mainly derived from thecal capillaries in the ovary cortical and granulosa cells secretion (30, 31), as well as oocytes. FF consists of hormones, electrolytes, anticoagulants, enzymes, reactive oxygen species, and antioxidants, which act as communicative mediators between oocytes and cumulus cells and mural granulosa cells, which are essential for female fertility (29). Except for the above components, our results showed that amino acids, carbohydrates, cofactors, vitamins, nucleosides, xenobiotics, and their derivates are also included in FF. Furthermore, we revealed that FF from OHSS patients showed 59 metabolites variation and 16 differentially expressed metabolites (8 increased metabolites including N-acetylornithine, mannitol, deoxyinosine, guanosine, L-isoleucine, pyruvic acid, maleamate, and arachidonic acid and other 8 decreased metabolites including L-2-hydroxyglutaric acid, L-carnitine, creatinine, putrescine, isovaleric acid, creatine, 4-hydroxyphenylacetaldehyde, and deoxycorticosterone in FF from OHSS patients) could be defined as potential diagnostic and treatment markers of OHSS.

Mannitol, widely used as a plasma expander, has been proven to reduce rates of moderate and severe OHSS in women at high risk of OHSS (32). For mannitol group (n=110), mannitol was injected daily from the day after the hCG injection until the third day after embryo transfer, 31 patients showed moderate or severe OHSS; for the control group (n=116), 60 out of the 116 women showed moderate or severe OHSS. However, in our study, the results showed that OHSS patients had higher mannitol levels in the FF compared with control patients, indicating that mannitol may be involved in the prevention of OHSS as a feedback regulator. Although mannitol had been reported to inhibit VEGF expression in rat vascular smooth muscle cells (33), whether mannitol reduces OHSS by regulating VEGF production in human granulosa cells is still largely unknown. For mannitol, how it is regulated in OHSS patients still needs to be further investigated. Guanosine, a purine nucleoside, can be phosphorylated to form guanosine monophosphate (GMP), cyclic GMP (cGMP), guanosine diphosphate (GDP), and guanosine triphosphate (GTP), which are mediators in signal transduction pathways. Guanosine is released from neuronal cells under pathological conditions to reduce inflammation and oxidant stress (34). It had been reported that E2 treatment could induce cGMP levels in immature *rat* uterine (35), and vice versa, cGMP analogue 8-bromo-guanosine 3′, 5′-cyclic monophosphate (8-br-cGMP) could also increase E2 expression in human ovary tissue, leading to an increasing proportion of secondary follicles (36). We first showed that guanosine was significantly higher in the FF of OHSS patients, and guanosine might also be used as one of the predictors of OHSS.

Lipid disorder is associated with OHSS pathology. A current retrospective analysis concludes that lipid metabolism affects the morbidity of OHSS and females with dyslipidemia are more likely to develop to OHSS (37). Other two studies demonstrate that various lipids altered in FF of OHSS patients (22, 38), indicating the significance of lipid metabolism on OHSS. Arachidonic acid (AA), an unsaturated and essential fatty acid, is produced from the phospholipids are catalyzed by phospholipase A2 (PLA2) and phospholipase C (PLC). Under inflammatory response, the free AAs were released (39–41). COS is regarded as an inflammatory statement (42). Furthermore, prostaglandin, a downstream metabolite of AA, was also reported to be involved in the occurrence of OHSS (43). More importantly, the attenuation of COX-2 decreases the incidence of OHSS in a rat model (44, 45). As the main fatty acid in FF (46), we found that the AA level in the FF of OHSS women was about 3 times higher than that of control patients, which is consistent with a current investigation (38). Moreover, our correlation analysis results showed that the higher AA was positively correlated with estradiol and No. of oocytes retrieved, although the former did not reach statistical significance. Previous and our studies proved that AA participates in the establishment of and could be used as a treatment target of OHSS.

Arginine (Arg), an essential amino acid, is metabolized by different enzymes, including arginine decarboxylase, arginase, and nitric oxide synthases (NOS) to produce urea, ornithine, and nitric oxide (NO), respectively (47). L-arginine has been reported to decrease estradiol production in mid- and late-human corpus luteum slices (48). Creatine, a substance belonging to arginine and proline metabolism, plays a critical role in regulating energy metabolism by catalyzing the reversible phosphotransfer between creatine and phosphocreatine systems. hCG administration enhances FF creatine expression on immature mice model (49). Interestingly, our study revealed that OHSS patients have lower creatine in FF, and it was negatively correlated with E2; however, in the control group, the FF creatine was positively correlated with E2. Our results indicate that creatine may have a bidirectional regulation effect in the E2 production in the control and OHSS patients; however, the underlying molecular mechanism needs to be further examined. Creatinine is a metabolite of creatine (50). Several studies reported the dynamic expression of creatinine in the ovary; for instance, FF creatinine concentrations peak at metestrus in *buffalo*, but other studies showed that FF creatinine concentrations remain unchanged in *buffalo* during different stages of folliculogenesis (51, 52). In pathological conditions, serum creatinine levels of PCOS are identical to non-PCOS in the rat model (53). Being similar to creatine, creatinine levels in the FF of OHSS patients were significantly lower and negatively related with E2 in the serum.

Deoxycorticosterone is a C-21 steroid hormone synthesized by the adrenal gland (54). Deoxycorticosterone is a precursor for corticosteroids and has potential mineralocorticoid activity. Corticosteroids were reported to block the platelet-derived growth factor-induced VEGF expression, but desoxycorticosterone does not affect VEGF in human vascular smooth muscle cells (55). Corticosteroids also inhibited the expression of VEGF in hemangioma-derived stem cells (56). We first reported that OHSS patients have lower levels of deoxycorticosterone in the FF, which were also negatively correlated with E2 production. Our results indicate that deoxycorticosterone may be used as a new treatment target for OHSS in the future.

Taken together, in this study, 8 metabolites including N-acetylornithine, mannitol, deoxyinosine, guanosine, L-isoleucine, pyruvic acid, maleamate, and arachidonic acid were found to be higher in the FF of OHSS patients. Among them, deoxyinosine levels were found positively correlated with E2, L-isoleucine, pyruvic acid, maleamate, and arachidonic acid were found to be positively related with the No. of retrieved oocytes in OHSS patients. These differentially changed metabolites may be involved in the OHSS pathological process and can be taken as diagnostic biomarkers for OHSS; however, the detailed roles of these metabolites in OHSS remain to be elucidated. Furthermore, other 8 metabolites, including L-2-hydroxyglutaric acid, L-carnitine, creatinine, putrescine, isovaleric acid, creatine, 4-hydroxyphenylacetaldehyde, and deoxycorticosterone decreased in the FF of OHSS patients compared with the control group. Among them, 4-hydroxyphenylacetaldehyde, deoxycorticosterone, creatinine, and creatine were found to be negatively associated with serum E2 levels on hCG day, and 4-hydroxyphenylacetaldehyde, L-carnitine, isovaleric acid, L-2-hydroxyglutaric acid were negatively related with No. of oocytes retrieved in OHSS patients. The identified 12 differentially changed metabolites could be regarded as predictive or treatment markers for OHSS according to their AUC values, which provides important insights into the clinical management of OHSS. Moreover, large-size sample investigations are needed on FF metabolic analysis of OHSS in the future.

## Conclusions

In summary, we conducted this metabolism analysis in human FF to identify potential diagnostic biomarkers for OHSS. The varied compounds in FF included amino acids, lipids, carbohydrates, cofactors, vitamins, nucleosides, xenobiotics, and their derivatives. Some of these differentially changed metabolites were associated with serum estradiol levels on the hCG day; some correlated with No. of oocytes retrieved in the OHSS group, indicating the potential role of such metabolites in OHSS pathology. This study provides us with a better acknowledgment of OHSS FF changes, and it needs more investigations with extended samples to validate the detailed underlying mechanism between differentially changed metabolites and the known OHSS biomarker.

## Data availability statement

The original contributions presented in the study are included in the article/supplementary material. Further inquiries can be directed to the corresponding author.

## Ethics statement

The study received approval from the Ethics Committee of Scientific Research and Clinical Trial of the First Affiliated Hospital of Zhengzhou University. Ethical review number: 2022-KY-252. The patients/participants provided their written informed consent to participate in this study.

## Author contributions

All authors listed have made a substantial, direct, and intellectual contribution to the work and approved it for publication.

## References

[1] Blumenfeld Z. The ovarian hyperstimulation syndrome. Vit hormones (2018) 107:423–51. doi: 10.1016/bs.vh.2018.01.018 29544639

[2] Yan Z Weich HA Bernart W Breckwoldt M Neulen J. Vascular endothelial growth factor (VEGF) messenger ribonucleic acid (mRNA) expression in luteinized human granulosa cells in vitro. J Clin Endocrinol Metab (1993) 77(6):1723–5. doi: 10.1210/jcem.77.6.8263163 8263163

[3] McClure N Healy DL Rogers PA Sullivan J Beaton L Haning RV Jr.. Vascular endothelial growth factor as capillary permeability agent in ovarian hyperstimulation syndrome. Lancet (8917) 1994:235–6:344. doi: 10.1016/s0140-6736(94)93001-5 7913160

[4] Nastri CO Teixeira DM Moroni RM Leitão VM Martins WP. Ovarian hyperstimulation syndrome: pathophysiology, staging, prediction and prevention. Ultrasound obstetr gynecol (2015) 45(4):377–93. doi: 10.1002/uog.14684 25302750

[5] Usta T Oral E. Is the measurement of anti-müllerian hormone essential? Curr Opin obstetr gynecol (2012) 24(3):151–7. doi: 10.1097/GCO.0b013e3283527dcf 22487725

[6] Fang L Yan Y Wang S Guo Y Li Y Jia Q. High ovarian GDF-8 levels contribute to elevated estradiol production in ovarian hyperstimulation syndrome by stimulating aromatase expression. Int J Biol Sci (2021) 17(9):2338–47. doi: 10.7150/ijbs.60332 PMC8241723 34239360

[7] Cheng JC Fang L Li Y Wang S Li Y Yan Y. Melatonin stimulates aromatase expression and estradiol production in human granulosa-lutein cells: relevance for high serum estradiol levels in patients with ovarian hyperstimulation syndrome. Exp Mol Med (2020) 52(8):1341–50. doi: 10.1038/s12276-020-00491-w PMC8080626 32855437

[8] Fang L Li Y Wang S Li Y Chang HM Yi Y. TGF-β1 induces VEGF expression in human granulosa-lutein cells: a potential mechanism for the pathogenesis of ovarian hyperstimulation syndrome. Exp Mol Med (2020) 52(3):450–60. doi: 10.1038/s12276-020-0396-y PMC7156760 32152452

[9] Fang L Yu Y Li Y Wang S He J Zhang R. Upregulation of AREG, EGFR, and HER2 contributes to increased VEGF expression in granulosa cells of patients with OHSS†. Biol Reprod (2019) 101(2):426–32. doi: 10.1093/biolre/ioz091 31167229

[10] Cheng JC Fang L Chang HM Sun YP Leung PC. hCG-induced Sprouty2 mediates amphiregulin-stimulated COX-2/PGE2 up-regulation in human granulosa cells: a potential mechanism for the OHSS. Sci Rep (2016) 6:31675. doi: 10.1038/srep31675 27539669 PMC4990972

[11] Gonzalez-Covarrubias V Martínez-Martínez E Del Bosque-Plata L. The potential of metabolomics in biomedical applications. Metabolites (2022) 12(2):194. doi: 10.3390/metabo12020194 35208267 PMC8880031

[12] Pautova A Burnakova N Revelsky A. Metabolic profiling and quantitative analysis of cerebrospinal fluid using gas chromatography-mass spectrometry: Current methods and future perspectives. Mol (Basel Switzerland) (2021) 26(12):3597. doi: 10.3390/molecules26123597 PMC8231178 34208377

[13] Liu Y Mei B Chen D Cai L. GC-MS metabolomics identifies novel biomarkers to distinguish tuberculosis pleural effusion from malignant pleural effusion. J Clin Lab Anal (2021) 35(4):e23706. doi: 10.1002/jcla.23706 33528039 PMC8059743

[14] Beyoğlu D Simillion C Storni F De Gottardi A. Idle JR. a metabolomic analysis of cirrhotic ascites. Mol (Basel Switzerland) (2022) 27(12):3935. doi: 10.3390/molecules27123935 PMC9228447 35745058

[15] Chiu CY Lin G Cheng ML Chiang MH Tsai MH Lai SH. Metabolomic profiling of infectious parapneumonic effusions reveals biomarkers for guiding management of children with streptococcus pneumoniae pneumonia. Sci Rep (2016) 6:24930. doi: 10.1038/srep24930 27103079 PMC4840347

[16] Revelli A Delle Piane L Casano S Molinari E Massobrio M Rinaudo P. Follicular fluid content and oocyte quality: from single biochemical markers to metabolomics. Reprod Biol endocrinol: RB&E (2009) 7:40. doi: 10.1186/1477-7827-7-40 19413899 PMC2685803

[17] Marianna S Alessia P Susan C Francesca C Angela S Francesca C. Metabolomic profiling and biochemical evaluation of the follicular fluid of endometriosis patients. Mol Biosyst (2017) 13(6):1213–22. doi: 10.1039/C7MB00181A 28475193

[18] Cordeiro FB Cataldi TR de Souza BZ Rochetti RC Fraietta R Labate CA. Hyper response to ovarian stimulation affects the follicular fluid metabolomic profile of women undergoing IVF similarly to polycystic ovary syndrome. Metabolomics (2018) 14(4):51. doi: 10.1007/s11306-018-1350-z 30830356

[19] Chen J Zhou Q Zhang Y Tan W Gao H Zhou L. Discovery of novel serum metabolic biomarkers in patients with polycystic ovarian syndrome and premature ovarian failure. Bioengineered (2021) 12(1):8778–92. doi: 10.1080/21655979.2021.1982312 PMC8806610 34696698

[20] Liu R Bai S Zheng S Zhu X Zhang Y Xu B. Identification of the metabolomics signature of human follicular fluid from PCOS women with insulin resistance. Dis Markers (2022) 2022:6877541. doi: 10.1155/2022/6877541 35465261 PMC9019454

[21] Liang C Zhang X Qi C Hu H Zhang Q Zhu X. UHPLC-MS-MS analysis of oxylipins metabolomics components of follicular fluid in infertile individuals with diminished ovarian reserve. Reprod Biol endocrinol: RB&E (2021) 19(1):143. doi: 10.1186/s12958-021-00825-x 34521427 PMC8438979

[22] Gao Y Li J Fan S Chen P Huang M Bi H. Lipid analysis of follicular fluids by UHPLC-ESI-HRMS discovers potential biomarkers for ovarian hyperstimulation syndrome. Front Endocrinol (2022) 13:895116. doi: 10.3389/fendo.2022.895116 PMC9276923 35846297

[23] Shmorgun D Claman P. The diagnosis and management of ovarian hyperstimulation syndrome. J Obstet Gynaecol Can (2011) 33(11):1156–62. doi: 10.1016/S1701-2163(16)35085-X 22082791

[24] Golan A Weissman A. Symposium: Update on prediction and management of OHSS. a modern classification of OHSS. Reprod biomed online (2009) 19(1):28–32. doi: 10.1016/S1472-6483(10)60042-9 19573287

[25] Bijlsma S Bobeldijk I Verheij ER Ramaker R Kochhar S Macdonald IA. Large-Scale human metabolomics studies: a strategy for data (pre-) processing and validation. Anal Chem (2006) 78(2):567–74. doi: 10.1021/ac051495j 16408941

[26] Navot D Bergh PA Laufer N. Ovarian hyperstimulation syndrome in novel reproductive technologies: prevention and treatment. Fertility sterility (1992) 58(2):249–61. doi: 10.1016/s0015-0282(16)55188-7 1633889

[27] Aboulghar M. Prediction of ovarian hyperstimulation syndrome (OHSS). estradiol level has an important role in the prediction of OHSS. Hum Reprod (Oxford England) (2003) 18(6):1140–1. doi: 10.1093/humrep/deg208 12773437

[28] Ocal P Sahmay S Cetin M Irez T Guralp O Cepni I. Serum anti-müllerian hormone and antral follicle count as predictive markers of OHSS in ART cycles. J Assisted Reprod Genet (2011) 28(12):1197–203. doi: 10.1007/s10815-011-9627-4 PMC3241835 21882017

[29] Basuino L Silveira CF Jr. Human follicular fluid and effects on reproduction. JBRA assisted reproduction (2016) 20(1):38–40. doi: 10.5935/1518-0557.20160009 27203305

[30] Hennet ML Combelles CM. The antral follicle: a microenvironment for oocyte differentiation. Int J Dev Biol (2012) 56(10-12):819–31. doi: 10.1387/ijdb.120133cc 23417404

[31] Rodgers RJ Irving-Rodgers HF. Formation of the ovarian follicular antrum and follicular fluid. Biol Reprod (2010) 82(6):1021–9. doi: 10.1095/biolreprod.109.082941 20164441

[32] Youssef MA Mourad S. Volume expanders for the prevention of ovarian hyperstimulation syndrome. Cochrane Database sys Rev 2016(8):Cd001302. doi: 10.1002/14651858.CD001302.pub3 PMC9243766 27577848

[33] Dulak J Tomala K Loboda A Józkowicz A. Nitric oxide-dependent synthesis of vascular endothelial growth factor is impaired by high glucose. Life Sci (2004) 75(21):2573–86. doi: 10.1016/j.lfs.2004.05.021 15363662

[34] Bettio LE Gil-Mohapel J Rodrigues AL. Guanosine and its role in neuropathologies. Purinergic signal (2016) 12(3):411–26. doi: 10.1007/s11302-016-9509-4 PMC5023624 27002712

[35] Alexandre K Rooryck J Galand P. Dissociation by colchicine of the wet weight and the cGMP responses to estradiol in immature rat uterus. J Steroid Biochem (1988) 31(6):873–5. doi: 10.1016/0022-4731(88)90327-5 2848986

[36] Scott JE Zhang P Hovatta O. Benefits of 8-bromo-guanosine 3’,5’-cyclic monophosphate (8-br-cGMP) in human ovarian cortical tissue culture. Reprod biomed online (2004) 8(3):319–24. doi: 10.1016/S1472-6483(10)60912-1 15038897

[37] Liu F Jiang Q Sun X Huang Y Zhang Z Han T. Lipid metabolic disorders and ovarian hyperstimulation syndrome: A retrospective analysis. Front Physiol (2020) 11:491892. doi: 10.3389/fphys.2020.491892 33329009 PMC7711040

[38] Sun Y Hao L Han W Luo J Zheng J Yuan D. Intrafollicular fluid metabolic abnormalities in relation to ovarian hyperstimulation syndrome: Follicular fluid metabolomics *via* gas chromatography-mass spectrometry. Clin chim Acta (2023) 538:189–202. doi: 10.1016/j.cca.2022.11.033 36566958

[39] van Dorp DA. Essential fatty acid metabolism. Proc Nutr Society (1975) 34(3):279–86. doi: 10.1079/PNS19750050 813233

[40] Sperling RI Benincaso AI Knoell CT Larkin JK Austen KF Robinson DR. Dietary omega-3 polyunsaturated fatty acids inhibit phosphoinositide formation and chemotaxis in neutrophils. J Clin Invest (1993) 91(2):651–60. doi: 10.1172/JCI116245 PMC288002 8381824

[41] de Jonge HW Dekkers DH Lamers JM. Polyunsaturated fatty acids and signalling via phospholipase c-beta and A2 in myocardium. Mol Cell Biochem (1996) 157(1-2):199–210. doi: 10.1007/BF00227899 8739247

[42] Orvieto R. Controlled ovarian hyperstimulation–an inflammatory state. J Soc Gynecol Invest (2004) 11(7):424–6. doi: 10.1016/j.jsgi.2004.05.001 15458738

[43] Schenker JG Polishuk WZ. The role of prostaglandins in ovarian hyperstimulation syndrome. Eur J obstetr gynecol Reprod Biol (1976) 6(2):47–52. doi: 10.1016/0028-2243(76)90001-0 985762

[44] Quintana R Kopcow L Marconi G Young E Yovanovich C Paz DA. Inhibition of cyclooxygenase-2 (COX-2) by meloxicam decreases the incidence of ovarian hyperstimulation syndrome in a rat model. Fertility steril (2008) 90(4 Suppl):1511–6. doi: 10.1016/j.fertnstert.2007.09.028 18166186

[45] Kitsou C Kosmas I Lazaros L Hatzi E Euaggelou A Mynbaev O. Ovarian hyperstimulation syndrome inhibition by targeting VEGF, COX-2 and calcium pathways: a preclinical randomized study. Gynecol Endocrinol (2014) 30(8):587–92. doi: 10.3109/09513590.2014.910191 24819316

[46] Liu J Brown RE. Immunohistochemical expressions of fatty acid synthase and phosphorylated c-met in thyroid carcinomas of follicular origin. Int J Clin Exp pathol (2011) 4(8):755–64. PMC3225787 22135723

[47] Wu G Bazer FW Davis TA Kim SW Li P Marc Rhoads J. Arginine metabolism and nutrition in growth, health and disease. Amino Acids (2009) 37(1):153–68. doi: 10.1007/s00726-008-0210-y PMC2677116 19030957

[48] Vega M Urrutia L Iñiguez G Gabler F Devoto L Johnson MC. Nitric oxide induces apoptosis in the human corpus luteum in vitro. Mol Hum Reprod (2000) 6(8):681–7. doi: 10.1093/molehr/6.8.681 10908276

[49] Umehara T Kawai T Goto M Richards JS Shimada M. Creatine enhances the duration of sperm capacitation: a novel factor for improving *in vitro* fertilization with small numbers of sperm. Hum Reprod (Oxford England) (2018) 33(6):1117–29. doi: 10.1093/humrep/dey081 PMC5972610 29635630

[50] Price CP Finney H. Developments in the assessment of glomerular filtration rate. Clin chim Acta (2000) 297(1-2):55–66. doi: 10.1016/S0009-8981(00)00233-3 10841908

[51] Tabatabaei S Mamoei M. Biochemical composition of blood plasma and follicular fluid in relation to follicular size in buffalo. Comp Clin Pathol (2011) 20(5):441–5. doi: 10.1007/s00580-010-1014-5

[52] Abd Ellah M Hussein H Derar D. Ovarian follicular fluid constituents in relation to stage of estrus cycle and size of the follicle in buffalo. Vet World (2010) 3(6):263–7.

[53] Sadrefozalayi S Farokhi F. Effect of the aqueous extract of foeniculum vulgare (fennel) on the kidney in experimental PCOS female rats. Avicenna J phytomed (2014) 4(2):110–7. PMC4103710 25050308

[54] Nahoul K Dehennin L Salat-Baroux J Scholler R. Deoxycorticosterone secretion by the human ovary. J Steroid Biochem (1988) 31(1):111–7. doi: 10.1016/0022-4731(88)90213-0 3398524

[55] Nauck M Karakiulakis G Perruchoud AP Papakonstantinou E Roth M. Corticosteroids inhibit the expression of the vascular endothelial growth factor gene in human vascular smooth muscle cells. Eur J Pharmacol (1998) 341(2-3):309–15. doi: 10.1016/S0014-2999(97)01464-7 9543253

[56] Greenberger S Boscolo E Adini I Mulliken JB Bischoff J. Corticosteroid suppression of VEGF-a in infantile hemangioma-derived stem cells. New Engl J Med (2010) 362(11):1005–13. doi: 10.1056/NEJMoa0903036 PMC2845924 20237346

